# Account of an Organic Disease of the Heart

**Published:** 1809-08

**Authors:** M. Boucher

**Affiliations:** Surgeon at La Fleche


### *Account of an Organic Disease of the Heart*; communicated by M. Boucher, Surgeon at La Fleche.*

Mademoiselle R——, born of strong and healthy parents, enjoyed a good state of health until she was fifteen years of age, when she was attacked by a putrid fever, produced in all probability by an irregular diet; from this attack she completely recovered.

During a year she increased in strength, and grew taller: every thing, so far as age or health were concerned, gave tokens that her menses would soon appear. She became pale, however, and bloated, and soon complained of oppression of the chest, and palpitation. I thought these symptoms were as much to be ascribed to the effects of chlorosis as to worms; and consequently prescribed martial tinctures and anthelmintics. I confess I felt considerable uneasiness, when I found that the palpitation continued night and day. When asleep she threw off the bed clothes without awakening; a symptom not observable in an affection purely nervous. Besides, the palpitation was not clear and distinct, but tumultuous and confused, and never in unison with the vibration of the pulse.

Menstruation at length commencing, her friends thought all danger at an end: for my own part, my satisfaction was only temporary; the bloatedness of the countenance re-appeared, the lips became of a livid hue: the tip of the nose assumed the same colour, as well as the centre of the cheeks; the eyes were a little swollen. Having felt the region of the precordium, I perceived the same tumultuous motion. I communicated to her friends my fears, with respect to the defective organization in the source of circulation.

At this period, a second putrid fever attacked the patient, and after having brought her to the brink of the grave, it yielded to the usual remedies. Her recovery on this occasion gave her friends reason to hope that the whole system had undergone a change, which could not fail to establish her health in future. I could not be persuaded, however, of her complete recovery, having always observed the same palpitation of the heart: the bloated appearance of her skin had of course ceased, but

* From the Journal de Medicine.

this frequently proceeds from diet, blisters, and universal debility: the paleness of the skin, the necessary consequence of this fever, had banished the livid colour of the countenance. I thought it most prudent to keep up a discharge from the legs, and to revert to the use of anthelmintics and emmenagogues.

The patient's growth had increased during the disease, and her figure was now tall, inclining to *embon point*, her limbs became plump, and her breasts swelled; her countenance became agreeable; her courses were regular, and, though not abundant, they were unaccompanied with any painful sensations; her dispositions were gentle, inclining to gaiety. In the midst of all these natural advantages, I witnessed the same symptoms which had formerly given me so much uneasiness.

I then consulted two eminent practitioners in the capital. It was their joint opinion that the patient's case was a severe chlorosis, the consequence of a general atony, and particularly of the uterus, which gave reason to apprehend an organical disorder in the heart, or in its large vessels, if the elasticity of the fibres was not speedily increased. Aperients and emmenagogues under different forms were prescribed.

I did not exactly coincide with this opinion; 1st. Because no hysteric symptom had ever taken place, and it seemed to them, that the womb or its appendages were affected, rather than the heart and its large vessels: besides, young boys are also subject to this dreadful disease.

2dly, Because I thought the organical defect already existed. I adhered however to the prescriptions, even not omitting the vomits which I was desired to administer from time to time.

The patient took several of these remedies, but, like all other persons similarly situated, she lost all confidence in them. M. Lepine, a physician, was then called in, and he agreed in opinion with me.

In this disagreeable situation, our patient was seized with a shivering, followed by fever. I was again sent for, and on examining found her heart in the same state, but beating more tumultuously; her face and limbs were bloated, and the violet colour was deeper.

Third day, An erysipelatous eruption appeared on the left leg; spirituous applications were employed.

Fifth day, Gangrenous spots appeared upon the foot: cinquona and generous wine were administered.

Eighth day, The alvine secretions were similar to those of of the *melæna*. I remarked that all postures were alike to the patient; she generally, however, lay on the left side.

Ninth day, Apparently improved throughout the whole system.

Eleventh day, The oppression, anxiety, and tumultuous motion of the heart were increased; the pulse was obliterated, an icy coldness covered the whole surface of the body; and she was at times delirious.

This state of agony having lasted four days, slight convulsions came on, and the patient died at the age of 25, having been ill about five years.

### Appearances on Dissection.

It was with some difficulty that I obtained leave to open the body, and I was only permitted to examine the heart. A confidential person was appointed to be present, and I was limited with respect to time.

Having raised the sternum, the pericardium presented itself like a large bladder, occupying a third of the cavity of the thorax, having pushed the lungs aside both behind and before. On opening it, about a wine glass of watery fluid escaped; the heart when exposed to view seemed to be two-thirds more than its ordinary size; the aorta and the pulmonary artery had preserved their natural form and size: the two auricles presented an extent proportioned to that of the heart; throughout they preserved their relative proportions; thus, the right auricle was larger and firmer than the left, and the colour deeper; we found a large glass full of blood in it, part of which was fluid, and part polypous; this last substance strongly adhered to, and was spread over the various curves and apertures of the heart. The tricuspid valves were five or six lines in length. The aperture by which this auricle pours the blood into the right ventricle would have admitted of a hen's egg.

The left auricle contained a smaller quantity of blood.

The scalpel, so far from shewing a tenuity in the ventricles, occasioned by a stretching of the organ, exhibited a thickness and consistence double that of Nature, particularly in the left ventricle; both contained a prodigious quantity of blood, partly fluid, and partly polypous: the latter strongly adhered to the curves and flexures of these organs: the left contained less than the right.

The lungs were soft and much inflated: the liver large and pale. To conclude, we perceived no particular affection in any other organ.

REFLECTIONS.—If the celebrated Corvisart and the most enlightened professional men, have not ventured to determine the efficient cause of organic defects in the heart, it would be highly presumptuous in me to hazard a definition. Besides this efficient cause always pre-supposes an original defect in the organization of the parts; thus, individuals are born with the lungs, the brain, or any other of the viscera so formed, as to produce mortal diseases sooner or later.

It is much easier to explain the symptoms. It is in fact easy to conceive that the veins have always the facility in every state of the body and lungs, of pouring the blood into capacious ventricles; and on the contrary, if the pulmonary arteries and the aorta are not of a proportionable calibre, the blood cannot leave the heart without difficulty, and not by a single *jet*: hence this perpetual tumultuous motion of the heart, the irritation being constant from the presence of this improper quantity of blood.

Nevertheless, although the apertures of the auricles were very large, from the venous blood of the heart filling them, they would become, (if I may use the expression) choaked, and not be perfectly emptied; particularly if, as in this case, the veins did not possess any contractile power. All these evils would therefore extend not only to the blood vessels, but also to the lymphatics, whence resulted a small menstrual discharge, a livid colour where these vessels abounded, filtrations through the cellular texture of the parts removed from the source of circulation, and a great difficulty of breathing; finally, the extinction of life, occasioned by the increase of debility, the polypous thickening of the blood in the ventricles, and the whole train of symptoms attending the case in question.

